# Perioperative Nursing of Patients with Reoperation of Recurrent Parathyroid Carcinoma Invading the Upper Digestive or Respiratory Tract

**DOI:** 10.1155/2020/6946048

**Published:** 2020-02-20

**Authors:** Lingxue Yin, Shui Feng, Zengxia Shi

**Affiliations:** Department of Otolaryngology Head and Neck Surgery, Beijing Shijitan Hospital Affiliated to Capital Medical University, Beijing, China

## Abstract

**Objective:**

The aim of this study was to summarize the perioperative nursing care of patients with recurrent parathyroid carcinoma.

**Methods:**

A retrospective analysis of 10 patients with recurrent parathyroid carcinoma was performed. The clinical data, diagnosis, treatment process, and nursing process (including clinical nursing intervention of various complications) were analyzed. The nursing experience and methods were discussed, summarized, and analyzed.

**Results:**

A total of 10 patients were reviewed (male : female 7 : 3; aged 48.6 ± 14.60 years). The mean interval between the initial operation and reoperation was 2.23 ± 1.65 years. The mean number of operations was 4.00 ± 1.41. Invasion of the trachea or esophagus was evident in 7 patients, larynx in 6 patients, recurrent laryngeal nerve in 1 patient, and cyclic cartilage in 2 patients. Serum calcium range was 3.20–4.68 mmol/L, and parathyroid hormone (PTH) range was 860–2830 pg/ml at admission. 6 patients underwent prophylactic tracheostomy, 2 patients underwent partial laryngectomy, and 2 patients underwent total laryngectomy. 1 patient experienced temporary water-electrolyte disorder and hypoproteinemia. The median serum calcium was 2.28 mmol/L (1.66–3.18 mmol/L) and median PTH level was 82.60 pg/ml (63.70–900.00 pg/ml) postoperatively. Serum PTH and calcium were still higher than the upper limit of normal in 2 patients after surgery. 2 of the other 8 patients relapsed within 8–11 months, and 6 patients remained normal for 11–40 months.

**Conclusion:**

For patients with reoperation of recurrent parathyroid carcinoma, high-quality, reasonable, and careful perioperative nursing ensured a successful operation and optimized outcome.

## 1. Introduction

Parathyroid carcinoma is a very rare cancer that accounts for only 0.05% of all cancers [1]. The clinical presentation of parathyroid carcinoma includes severe hypercalcemia, a palpable neck mass, an amino parathyroid hormone (PTH) ratio >1 as measured by the third/second PTH assay, and local invasion or regional metastasis. It is difficult to cure parathyroid carcinoma because of local recurrence or distant metastasis [2]. En-bloc resection surgery is the only reliable treatment for this disease [3, 4].

Most postoperative recurrence of parathyroid carcinoma occurs within 3 years after surgery [3, 5]. In some patients, the malignancy might invade the trachea, throat, esophagus, or pharynx. The difficulty of reoperation is obviously increased, due to scar tissue and complications including recurrent nerve injuries or trachea fistulas [6]. Despite these complications, surgery remains the most effective treatment [7]. Radiotherapy and chemotherapy have shown disappointing results for management of persistent, recurrent, or metastatic cases [8]. Currently, the goal of therapy for metastatic disease is to control the PTH-driven hypercalcemia which represents the primary cause of mortality [8].

The complicated condition of patients with recurrence and local invasion means that the scope of surgery is no longer to be curative, but to prolong survival. It is hoped that the hormone metabolism will be more controlled after operation if the tumor is completely removed [9]. However, removal of cancer that has invaded the respiratory tract can seriously affect the patient's respiratory condition. If the perioperative nursing care is not thorough, the surgical outcome will be seriously affected [10]. Guidelines for perioperative nursing care are not currently available.

In this study, we describe our perioperative nursing care for patients with recurrent parathyroid carcinoma invading the upper respiratory tract and digestive tract between 2006 and 2016 in our hospital. The aim is that these data could be used for perioperative nursing care services that may enable patients to be safely discharged from hospital (Supplementary [Supplementary-material supplementary-material-1]).

## 2. Methods

### 2.1. Patients

All patients with parathyroid carcinoma treated by otolaryngology head and neck surgery of Beijing Shijitan Hospital were retrospectively collected from January 2006 to December 2016. The patients were included in the study according to the following inclusion criteria: (1) age ≥18 years; (2) patients who had received at least one surgical treatment before admission; and (3) postoperative pathological diagnosis of recurrent parathyroid carcinoma and invasion of the upper digestive or respiratory tract. The exclusion criteria were as follows: (1) distant metastasis and (2) incomplete information.

This study was approved by the Ethics Committee of Beijing Shijitan Hospital Affiliated to Capital Medical University. Informed consent was waived because of the study's retrospective nature.

### 2.2. Preoperative Preparation

Preoperative monitoring was undertaken for electrolyte levels, blood pressure, and blood glucose. The patient's nervous system, gastrointestinal tract, and urinary tract symptoms were closely observed. Preoperative pressure sore assessment was performed using the Braden Scale, which measures elements of risk that contribute to either higher intensity and duration of pressure or lower tissue tolerance for pressure according to six subscales: sensory perception, moisture, activity, mobility, friction, and shear. Each item was scored between 1 and 4, with 1 having the most risk [11]. Signs of bone pain accompanied by polydipsia, polyuria, fatigue, and irritability were also monitored. For patients with hypercalcemia (especially those with high calcium crisis), according to the severity of the symptoms, hydration treatment, calcitonin, and dialysis treatment were extended.

The patients and their families were fully informed of the possible postoperative situation, and active cooperation of the family members was encouraged to reduce their anxiety. Hypocalcemia occurs after surgery, but any head and face numbness, extremity numbness, heart discomfort, and respiratory abnormalities should be promptly reported. Patients with tracheotomy will be unable to speak while the tracheotomy tube is in place and should prepare writing materials for communication. During nasal feeding, there will be discomfort such as abdominal distension, nausea, and low gustation, but the nurse can provide timely treatment. Patients with total laryngectomy will experience aphasia [12]. Patients with colonic esophageal reconstruction should be prepared to clean the intestine before surgery. The patient cannot eat after operation before the healing of the esophageal gastrointestinal anastomosis.

Precautionary measures were taken to prevent falls and inappropriate activities in patients who had a history of fractures. Special personnel accompanied the patients, activity was kept short, and patients were required to wear nonslip shoes. The floor was kept dry. A rail restraint was used on the patient's bed; appropriate walking aids or wheelchairs were provided.

Patients were asked to limit the intake of calcium and vitamin D in food before surgery, such as calcium-containing drugs, dairy products, eggs, fish, shrimps, and meat products, and reduce sun exposure. The patients were told to drink 2-3 L water a day to increase calcium in the urine.

A polyurethane gel position pad was placed at pressure sites during surgery to reduce the risk of pressure sores.

### 2.3. Postoperative Treatment

After operation, the respiratory rate, heart rate, bleeding, characteristics, and quantity of drainage fluid of the patients were observed closely. The PTH, serum calcium, and electrolyte levels were monitored according to the condition of the disease, and the corresponding treatment was carried out including personalized fluid supplement and calcium supplement.

The emotional changes of the patients and their families were observed. The nursing staff also mobilized family members to psychologically support the patient, preventing negative emotions.

After surgery, the nursing staff closely observed the wound for bleeding, errhysis, color, and elasticity of the skin. If extravasated blood and swollen skin were found, the patient may experience suffocation and severe pain. The doctor provided prompt treatment.

The drainage volume was recorded once every 24 hours. Generally, the drainage ball was removed when the drainage volume decreased below 10 mL and the fluid was yellow.

For patients with a tracheal tube, it was continuously lubricated with 0.9% physiological saline, and the inner cannula was cleaned three times a day. Sputum aspiration was performed, and the environment was kept moist. The tightness of the tracheal cannula was monitored to prevent pressure sores. Wound dressings were changed daily to prevent infection. Where the disease condition spread, the patient was instructed to eat and drink, and the tube was blocked occasionally to observe the patient's breathing.

For patients with an abdominal wound after colonic esophageal reconstruction, the appearance and exudation of the wound were closely observed; the color, traits, and volume of the abdominal drainage fluid were monitored. The bellyband was in place at least once a day and was tight without affecting the patient's respiratory activity. Continuous gastrointestinal decompression was performed during water deprivation.

Enteral nutrition was performed 24 h to 4 w after surgery. A normal diet was administrated according to the patient's condition. Milk products, eggs, fish, shrimps, and meat products were provided in enough quantities to increase the absorption of calcium.

The patients and family members were taught how to clean the tracheal cannula and prevent occlusion of the tracheal cannula. Showers and swimming were forbidden. The patient was advised to avoid activity in crowded public places, prevent colds, and seek timely medical advice when they experienced any discomfort.

### 2.4. Statistical Analysis

The SPSS 19.0 statistical software (IBM Corp., Armonk, NY, USA) was used for statistical analysis. Normally distributed continuous variables were expressed as means ± standard deviation (SD) and nonnormally distributed continuous variables were presented as median (range). The categorical variables were expressed as frequency and percentage.

## 3. Results

### 3.1. Baseline Characteristics

A total of 10 patients were enrolled in this study (Supplementary [Supplementary-material supplementary-material-1]). Baseline characteristics are shown as Table 1.

### 3.2. Preparation of Nursing Care

All 10 cases enrolled in this study had hypercalcemia. Among them, 8 patients had serum calcium of 3.78–4.68 mmol/L, which was considered as a high calcium crisis and was treated with hydration before operation. 5 patients received intravenous infusion of 2000 mL 0.9% NaCl and furosemide (20 mg/day). 3 patients received intravenous infusion of 2000 mL 0.9% NaCl and furosemide (20 mg/day), as well as intramuscular injection of 50–200 IU salmon calcitonin twice a day. 5 patients received hemodialysis every other day. The serum calcium of all 10 patients has decreased to 3.47–3.77 mmol/L after the preoperative nursing care prior to operation. Blood pressure was measured twice a day in 3 patients with hypertension. Blood glucose was measured 3 times a day in 1 person with diabetes mellitus. 2 patients with reflux esophagitis were given oral administration of domperidone (10 mg/day) or esomeprazole (20 mg/day) or famotidine 20 mg twice daily.

### 3.3. Surgical Procedures

Six patients underwent prophylactic tracheostomy and had an indwelling cannula; 2 patients underwent partial laryngectomy and had an indwelling tracheal cannula; and 2 patients underwent total laryngectomy and had an indwelling tracheal cannula (Table 2).

### 3.4. Postoperative Observation

Postoperative pathology showed invasion of the trachea or esophagus in 7 patients, larynx in 6 patients, recurrent laryngeal nerve in 1 patient, and cyclic cartilage in 2 patients.

Nine patients had numbness in the face or extremities within 24 hours after operation. Among them, 1 patient (Case No. 7) also had water-electrolyte disorder, liver and kidney dysfunction, and hypoproteinemia within 24 hours. After 13–40 days of treatment and care, the mean length of hospital stay was 26.10 ± 9.88 days; 9 patients had normal serum calcium, 8 patients had normal PTH; 1 case had PTH slightly higher than upper limit of normal (109.5 pg/mmol) and normal serum calcium; 1 case had PTH at their preoperative level and was treated with 0.9% sodium chloride injection 250 mL + pamidronate disodium injection 45 mg intravenous infusion for calcium reduction treatment. The median postoperative serum calcium level was 2.28 mmol/L (range 1.66–3.18 mmol/L). The median postoperative PTH level was 82.60 pg/mL (range 63.7–900 pg/mL). No adverse reactions occurred.

### 3.5. Postoperative Follow-Up

Six patients with tracheotomy were extubated within two weeks, 1 patient was extubated after 6 weeks, and 1 patient was extubated 3 months after operation. 2 cases with total laryngectomy carried the tube throughout the rest of their life.

Serum PTH and calcium were still higher than the upper limit of normal in 2 patients after surgery. 2 of the other 8 patients relapsed within 8–11 months, and 6 patients remained normal for 11–40 months.

Drugs were given for calcium-reducing treatment. The last follow-up was December 2018, and at this point, 2 patients were still alive.

## 4. Discussion

Appropriate nursing has a vital role in preventing adverse events during parathyroid carcinoma treatment [10]. For example, monitoring patients for hypocalcemia and active intervention are needed to prevent serious bone disease occurring [13]. In this study, we aimed to analyze and summarize the perioperative nursing care of patients with recurrent parathyroid carcinoma invading the upper digestive or respiratory tract. No adverse reactions occurred. We believe that the perioperative care of these patients played an important role in the success of surgery and in improving the prognosis of patients.

The perioperative nursing involved preoperative and postoperative nursing, but the focus for the two stages was different.

### 4.1. Preoperative Nursing Care

The success of the initial operation is the most important factor in parathyroid cancer prognosis [9]. The chance of a radical cure is significantly reduced after the first treatment regimen and the risk of surgery is significantly increased [6]. Therefore, mental anxiety for patients prior to surgery is likely to be high. Most of these patients also had fatigue, bone pain, urinary stones, and other serious discomforts which may lead them to lose confidence in treatment [14, 15]. An important aspect of preoperative nursing was resolving patient anxiety, increasing confidence in the fight against disease, and encouraging cooperation with preoperative examination and treatment.

Hypercalcemia occurred in all patients in this study. Serum calcium >3.77 mmol/L means patients are prone to parathyroid crisis, and the mortality rate can be very high [16]. Hypercalcemia can lead to neuropsychiatric symptoms such as hallucinations and delusions [17]. Heart rate, heart rhythm, blood pressure, respiration, and skin changes were closely observed. During the calcium-reducing treatment, the nursing staff monitored psychosomatic manifestations and prevented low potassium and low magnesium. Serum calcium levels in high-risk patients are preferably monitored every 3 hours, adjusting the infusion rate and maintaining electrolyte balance [18–20].

### 4.2. Postoperative Nursing Care

Reoperation for parathyroid carcinoma increases the risk of surgery. Parathyroid glands are difficult to locate due to their position and small size and their anatomical relationship with adjacent organs. The arteries and veins of the neck are some of the most important blood vessels in the whole body. This makes bleeding and wound monitoring an important aspect of postoperative nursing. The degree and color of drainage fluid was closely monitored alongside prevention of drainage clogging and a subcutaneous hematoma. Bright red drainage fluid may indicate bleeding from the wound.

The patients underwent a long surgical procedure which increased the risk of pressure sores [21]. Therefore, active postoperative monitoring was important. Tracheotomy nursing involved ensuring that the tracheal tube was unobstructed.

In this group of patients, the PTH values reduced and serum calcium began to decrease 10 hours after operation. The PTH decrease suggests effective removal of the parathyroid carcinoma [9]. But serum calcium reduction can result in serious bone disease. Osteoporosis or fibrous cystic osteitis are important features of hyperparathyroidism caused by parathyroid cancer, but postoperatively “bone starvation” is frequent [13]. Therefore, prevention requires close observation of serum calcium changes and prompt treatment. In order to correct hypocalcemia after surgery, patients were encouraged to eat high-calcium products in frequent small meals with high protein, high vitamin, and less bulk. Nasal feeding was commonly used because of its simplicity, economy, and convenience [22]. However, complications such as tube blockage, diarrhea, constipation, and aspiration pneumonia may occur [23]. The patient's response after eating was observed to prevent these complications.

The limitations of this study include the small sample size of the patients enrolled from one center. This was a retrospective analysis of the nursing care and no comparison group was available, so the effectiveness of the nursing provision could not be quantified.

In conclusion, we believe that the perioperative nursing care of patients who underwent reoperation of parathyroid cancer played an important role in the success of surgery and in improving the prognosis of patients. Firstly, because the conditions of the patients were complicated and the tumor invasion area was wide, the difficulty of surgery and the scope of surgery were increased. The nursing care was often a multiorgan and multisystem comprehensive treatment. Patients inevitably experienced great psychological pressure. So, we preoperatively treated patients with targeted psychological care to improve the negative emotions of the patients, which enhanced their confidence in surgery and greatly improved their cooperation with treatment. Secondly, vital signs before and after surgery, especially respiratory function and electrolyte levels, varied greatly and had a great impact on the patients. In this regard, we performed close monitoring of hormones and electrolyte levels in the body before and after surgery, dietary guidance, and calcium limitation or supplementation. The results showed that this was effective, and the clinical results were satisfactory. Thirdly, the requirements for nursing staff were relatively high. It was especially important to be good at observation with skilled understanding of the disease and the focus of care.

## 5. Limitations

The limitations of this study include the small sample size of the patients enrolled from one center. This was a retrospective analysis of the nursing care and no comparison group was available, so the effectiveness of the nursing provision could not be quantified.

## 6. Conclusion

In conclusion, we believe that the perioperative nursing care of patients who underwent reoperation of parathyroid cancer played an important role in the success of surgery and in improving the prognosis of patients. Firstly, because the conditions of the patients were complicated and the tumor invasion area was wide, the difficulty of surgery and the scope of surgery were increased. The nursing care was often a multiorgan and multisystem comprehensive treatment. Patients inevitably experienced great psychological pressure. So, we preoperatively treated patients with targeted psychological care to improve the negative emotions of the patients, which enhanced their confidence in surgery and greatly improved their cooperation with treatment. Secondly, vital signs before and after surgery, especially respiratory function and electrolyte levels, varied greatly and had a great impact on the patients. In this regard, we performed close monitoring of hormones and electrolyte levels in the body before and after surgery, dietary guidance, and calcium limitation or supplementation. The results showed that this was effective, and the clinical results were satisfactory. Thirdly, the requirements for nursing staff were relatively high. It was especially important to be good at observation with skilled understanding of the disease and the focus of care.

## Figures and Tables

**Table 1:** Baseline data of the 10 patients with recurrent parathyroid carcinoma.

Variables	
Age, years (mean ± SD)	48.60 ± 14.60
Male, *n* (%)	7 (70%)
Diabetes, *n* (%)	2 (20%)
Hypertension, *n* (%)	2 (20%)
Heart disease, *n* (%)	1 (10%)
Position of initial occurrence in the parathyroid gland, *n* (%)	
Left	3 (30%)
Upper	5 (50%)
Unknown	2 (20%)
Interval between the initial surgery and this surgery, years (mean ± SD)	2.23 ± 1.65
Average number of surgeries, times (mean ± SD)	4.00 ± 1.41
Preoperative serum calcium, mmol/L, median (range)	4.16 (3.20–4.68)
Preoperative PTH, pg/mL, median (range)	1954.75 (860–2830)

PTH: parathyroid hormone.

**Table 2:** Surgical information of the 10 cases with recurrent parathyroid carcinoma.

Variables	
Trachea, *n* (%)	
Prophylactic tracheostomy and indwelling cannula	6 (60%)
Partial laryngectomy and indwelling tracheal cannula	2 (20%)
Total laryngectomy and indwelling tracheal cannula	2 (20%)
Postoperative serum calcium, mmol/l, median (range)	2.28 (1.66–3.18)
Postoperative PTH, pg/ml, median (range)	82.60 (63.70–900.00)

PTH: parathyroid hormone.

## Data Availability

The datasets used and/or analyzed during the current study are available from the corresponding author upon reasonable request.

## Supplementary Materials

Supplementary Materials Supplementary Table 1. Clinical characteristics of the ten cases who underwent reoperation of recurrent parathyroid carcinoma. Supplementary Table 2. Clinical nursing flow chart.
