# Supplementary material for: Perioperative Nursing of Patients with Reoperation of Recurrent Parathyroid Carcinoma Invading the Upper Digestive or Respiratory Tract
Source: Biomed Res Int. 2020 Feb 20;2020:6946048. doi: 10.1155/2020/6946048 (PMC7054761; doi:10.1155/2020/6946048)
Supplement: Supplementary Materials — Supplementary Table 1. Clinical characteristics of the ten cases who underwent reoperation of recurrent parathyroid carcinoma. Supplementary Table 2. Clinical nursing flow chart. [file 6946048.f1.pdf]

Supplementary Table 1 Clinical characteristics of the ten cases who underwent reoperation of recurrent parathyroid carcinoma

Case no.	Gender	Age	Number of surgeries	Position of relapse	Renal injury	Osteitis fibrosa cystica	Surgical range this time	Postoperative complications
1	Male	38	7	Larynx	Present	Present	Resection of recurrent parathyroid carcinoma and residual thyroid, partial laryngectomy and tracheotomy	Hypocalcemia
2	Male	40	4	Right tracheoesophageal groove, the VI and VII lymph nodes	Present	Present	Resection of recurrent parathyroid carcinoma and right thyroid, partial trachea-esophagectomy and repair, cervical lymph node dissection and tracheotomy	Hypocalcemia
3	Female	78	4	Left tracheoesophageal groove, the seventh cervical vertebra	Present	Present	Resection of recurrent parathyroid carcinoma and left thyroid, cervical lymph node dissection, and the local expansion and resection of the C7 vertebral fascia and Tracheotomy	Hypocalcemia
4	Male	58	5	Left tracheoesophageal groove, larynx and hypopharynx	Present	Present	Resection of recurrent parathyroid carcinoma and left thyroid, tracheotomy, partial trachea-esophagectomy and repair and cervical lymph node dissection	Hypocalcemia
5	Female	34	2	Larynx	Absent	Absent	Recurrent parathyroid carcinoma, tracheotomy, partial trachea-esophagectomy and repair, and cervical lymph node dissection	Hypocalcemia
6	Female	47	3	Left tracheoesophageal groove, the VI and VII lymph nodes	Present	Absent	Resection of recurrent parathyroid carcinoma, partial trachea-esophagectomy and repair, cervical lymph node dissection and tracheotomy	Hypocalcemia
7	Male	34	4	Extensive trachea and esophagus, larynx, with cricoid cartilage invasion and the VI and VII lymph nodes metastasis	Present	Present	Recurrent parathyroid carcinoma, left thyroidectomy, total laryngectomy, cervical esophagectomy, bilateral cervical lymph node dissection, colonic esophageal reconstruction and tracheotomy	Hypocalcemia Hypophosphatemia Hypoproteinemia Liver and

								kidney dysfunction
8	Male	60	5	Cricoid cartilage plane, between the tracheal membrane and esophagus	Absence	Presence	Recurrent parathyroid carcinoma, bilateral thyroidectomy, total laryngectomy, partial hypopharyngeal esophagectomy, bilateral cervical lymph node dissection, colonic esophageal reconstruction and tracheotomy	Hypocalcemia
9	Male	59	3	Esophageal, laryngeal and lymph node			Left parathyroidectomy + bilateral neck dissection + paralaryngeal esophageal mass resection + tracheotomy	Hypocalcemia
10	Male	38	3	Laryngeal and III, IV, VI pretracheal lymph node			Parathyroidectomy + left thyroidectomy and isthmic resection + left neck dissection (III IV IV) + tracheotomy + left recurrent laryngeal nerve formation + left neck vascular exploration + scar lysis	Hypocalcemia

Supplementary Table 2 Clinical nursing flow chart

**Preoperative Preparation**	**Psychological Nursing**	✓ Explaining the knowledge of disease to patients by multimedia<br>✓ Adopting appropriate ways to conduct psychological counseling for patients<br>✓ Helping patients relieve negative emotions and building self-confidence<br>✓ Guiding family members' concern for patients<br>✓ Providing the good hospitalization environment
	**Hypercalcemia Nursing**	According to the severity, the patients with hypercalcemia (especially the patients with hypercalcemia crisis) were given the treatment of dilatation and hydration, diuretics, and calcitonin intramuscular injection. If necessary, they were given emergency hemodialysis. During the treatment, nurses should closely observe the changes of heart rate, heart rate, blood pressure, respiration and skin, adjust the infusion dropping speed in time, prevent the treatment of high calcium and low potassium and magnesium, and maintain the electrolyte balance. [12-14]Monitor blood pressure and blood sugar before operation, adjust and deal with them in time. Giving symptomatic treatment to patients with gastrointestinal diseases.
	**Life Care**	✓ Preventing patients' falls and inappropriate activities<br>✓ The nursing staff should take precautions and specially assigned person to accompany them. The activity time should not be long and the activity volume should not be large. The patients are required to wear antiskid shoes.<br>✓ Keeping the floor dry, applying bed guards, and providing walking aids or wheelchairs.
	**Diet Nursing**	Patients must limit the intake of calcium and vitamin D in food before the operation and reduce the sun exposure and calcium absorption. The nursing staff should remind the patients to drink more water, 2-3l per day, to increase the excretion of calcium in urine. For those who have reconstruction of the esophagus with colon, the basic intake of the body should be guaranteed while eating a low calcium diet.
	**Prevention of Operation Pressure Sore**	Preoperative pressure sore evaluation should be as detailed as possible, including anesthesia, nutrition, body type, skin condition, operation method and position[15]. The polyurethane gel position pad is applied to the pressure part of the patient during operation, which can maximize the dispersion of local pressure and avoid and reduce the pressure ulcer damage.

<table>
<tr><td rowspan="6">Postoperative Preparation</td><td>Mental Nursing</td><td colspan="2">Observing the emotional changes of patients and their families in time, telling the patients the decrease of PTH and blood calcium during the operation, the approximate time of hypocalcemia, and preventing negative emotions. Because the patients who fail in the operation will have the change of regret and depression, the nursing staff should analyze the causes in time, explain the consequences and follow-up treatment methods to the patients, and enhance their confidence.</td></tr>
<tr><td rowspan="4">Specialized Nursing After Operation</td><td>Monitoring PTH and Blood Calcium</td><td>Because the increase of PTH plays a decisive role in the diagnosis of hyperparathyroidism, the removal of PTH plays an accurate role in the diagnosis of hyperparathyroidism.</td></tr>
<tr><td>Nursing Care of Hypocalcemia</td><td>Patients were given 10% calcium gluconate intravenous rehydration (intravenous rehydration is to control the dropping speed, prevent cardiac arrest) or oral calcium and vitamin D calcium, and routine monitoring of PTH and blood calcium concentration.</td></tr>
<tr><td>Tracheostomy Care</td><td>It is necessary to ensure that the tracheal tube is unobstructed, use 0.9% saline for atomization inhalation every day, and clean and replace the inner tube three times a day; Timely use of sterile suction tube for patients; Keeping the environment moist; Pay attention to the tightness of the tether of the tracheal tube to prevent pressure sore at the disconnection and incision.
Appropriate use of antibiotics and daily dressing change of wounds
If the patient's condition allows, guide the patient to eat and drink water, plug the tube in time, and observe the patient's breathing.</td></tr>
<tr><td>If the patient's condition allows, encourage him to get out of bed and exercise in time</td><td>Get out of bed as soon as possible. If the patient's condition is not allowed, appropriate hook foot exercise can be done on the bed, which is conducive to the blood return of lower limbs. It is better to do 3 times / h, or passive exercise with the help of the patient, so as to prevent the formation of venous thrombosis.</td></tr>
<tr><td>General Nursing After Operation</td><td colspan="2">Closely observe the vital signs of patients, monitor the level of PTH blood calcium and electrolyte according to the condition, and carry out corresponding treatment, personalized rehydration and calcium therapy. Patients were given nasal feeding care.</td></tr>
</table>

	**Hemorrhage**	Closely observe the bleeding and bleeding of the wound, closely observe the color and elasticity of the skin at the wound, and inform the doctor in time to take decisive measures in case of congestion and swelling of the skin, suffocation, sharp pain and other discomfort.
	**Observation of Drainage Fluid**	✓ Nurses should carefully observe whether there is negative pressure in the drainage ball, the color and character of the drainage fluid, and the drainage flow.<br>✓ In order to prevent the drainage ball from blocking, nurses need to squeeze the drainage tube for patients regularly.<br>✓ Recording the induced flow every 24 hours.
	**Nursing of Abdominal Wound After Operation of Colon with Esophagus**	Nurses need to closely observe the appearance and exudation of patients' wounds, and observe the color, character and volume of abdominal drainage fluid.<br>The patient's abdominal belt should be bandaged at least once a day.<br>Patients must fast water 3-6 days after operation [20], during which continuous gastrointestinal decompression is required.
	**Diet nursing**	In order to correct the hypocalcemia, he was encouraged to eat high calcium products, and the absorption of calcium was increased.
**Discharge Guidance**	**Regulating Thyroid Function**	In order to prevent hypothyroidism, guide patients to take medicine correctly and avoid missing or misusing. One month later, the thyroid function of the patient was rechecked, and the dosage was adjusted in time to ensure the normal function of the body.
	**Calcium Supplement**	After operation, the patients must insist on calcium supplement, and supplement vitamin D on time. PTH and blood calcium should be reexamined regularly, the interval time is 1-6 months, monitoring life.
	**Nursing care of patients with tracheotomy after discharge**	✓ Helping the patients and their families to learn to clean the endotracheal tube<br>✓ In order to prevent the blockage of the tracheal tube, the patient should not shower or swim<br>✓ Patients need to be prevented from cold and seek medical treatment in time
